# Approach To The First Unprovoked Seizure- PART II

**Published:** 2013

**Authors:** Mohammad Ghofrani

**Affiliations:** 1 Pediatric Neurology Research Center, Shahid Beheshti University of Medical Sciences, Tehran, Iran; 2 Pediatric Neurology Department, Mofid Children Hospital, Faculty of Medicine, Shahid Beheshti University of Medical Sciences, Tehran, Iran

**Keywords:** First, Unprovoked, Seizure, Children, Anti Epileptic Drugs (AED), Treatment

## Abstract

The approach to a child who has experienced a first unprovoked generalized tonic-clonic seizure is challenging and at the same time controversial.

How to establish the diagnosis, ways and means of investigation and whether treatment is appropriate, are different aspects of this subject.

In this writing the above mentioned matters are discussed.

## Introduction

**Risk factors for abnormal neuroimaging**

Children who were neurologically abnormal were more likely to have abnormal imaging. Children with partial seizure were more likely to have an abnormal imaging compared to children with generalized seizure. Children with status epilepticus and older children were more likely to be imaged but did not have an increased probability of abnormal imaging study.

Children who had abnormal imaging studies were more likely to experience further seizure during follow-up compared with children who had normal imaging studies. 

While the yield in finding an acute lesion is low, there appears to be substantial yield of imaging abnormalities in children with a first unprovoked seizure (22).

While most of these abnormalities may not change the immediate treatment, they do influence prognosis and may influence the decision of whether to treat or not to treat. In the Connecticut study, where 80% of children with newly diagnosed epilepsy were imaged, there was a statistically significant higher rate of imaging abnormalities in children with partial seizure and focal EEG finding, but this rate was still very low in children who were otherwise neurologically normal ( 31). Also, one study using high resolution MRI in randomly selected outpatients with epilepsy reports abnormalities in 50%, with mesial temporal sclerosis and heterotopia being the most common (31). Given the importance of neuroimaging in defining etiology and prognosis, as well as in influencing the decision of whether or not to initiate antiepileptic drug therapy, it would appear to be a rational diagnostic test even in children with a first unprovoked seizure, particularly in those cases where sedation is not necessary (32).

As MRI is clearly superior to CT as an imaging modality in this clinical setting, it would be reasonable in most cases to schedule MRI nonemergently rather than perform a CT in the emergency department and then also try and get an MRI later (22). A more difficult question to answer is whether or not to routinely image after the first seizure. Should children with a first unprovoked tonoclonic seizure whose neurological examination is normal and his/her seizure was of short duration be imaged or imaging be restricted to those who experience a recurrence (33). Also, it should be known MRI of the brain provides the most detailed and helpful anatomic information. This test is strongly recommended after certain electroencephalogram findings such as focal slowing or absence of electrographic pattern consistent with a benign epilepsy or primary generalized epilepsy. 

If a temporal lobe lesion is under consideration, it is necessary to have this imaging cut through the temporal lobes. Usually, the MRI is not a part of the workup in the emergency department but as part of a comprehensive outpatient evaluation (1).

In conclusion of this chapter, regarding the advisability of performing MRI imaging after first unprovoked seizure, the probability of finding an abnormality on neuroimaging requiring immediate medical or surgical intervention in children who experience first unprovoked seizure is approximately 1%. However, even children with a normal neurological examination, a generalized short seizure and a normal EEG, have a substantial probability of an abnormal imaging study. These abnormalities are relevant to prognosis and management. Imaging with the current state of knowledge about MRI may show a significant proportion of imaging abnormalities in children who seemingly had cryptogenic seizures (22). 

## EEG in first unprovoked seizure

EEG is extremely useful for clarifying seizure type, aiding in correct classification, and assisting in predicting long-term prognosis. It is also helpful in recognition of subclinical seizure, abnormal metabolic states and different encephalopathic conditions. Except in rare cases, there is no reason to obtain an EEG on emergency basis, but it should be performed soon after the event. Indeed, postictal EEG findings have predictive value. Having EEG, preferably obtained within few days after a seizure, may reveal focal or lateralized abnormalities that may be absent later. EEG should be obtained while the patient is awake, drowsy and asleep. 

Having EEG while utilizing different activating systems like hyperventilation, photic stimulation and obtaining sleep deprived EEG may increase the yield for detection of abnormalities (1).

## How likely is another seizure after the first unprovoked one?

The cumulative risk of recurrence increases over time; however, in studies where information is available, the majority of recurrences occur within the first 1 to 2 years (2, 34-37). At any given time, the reported risk of recurrence is highly variable. For example, at 1 year it ranges from a low of 14% to a high of 65% (38). 

Methodologic differences in seizure identification, age ranges and follow up of study participants, may also contribute to this variability (2).

## How often are children who present after first unprovoked seizure going to have multiple recurrences?

A minority of children will go on to experience not just one but many recurrences. One study that enrolled 207 children with follow-up for 2 years, found that in addition to an overall recurrence rate of 54%, 26% of the enrolled children were still experiencing one or more seizures during the last 6 months of the study follow up, that is, over18 months after the index event (36). Anotherstudy with longer follow up, enrolled 407 children and followed them for an average of > 10 years. Of these, 46% had one or more recurrences during that period of time. Over the extended follow up period, 19% of the children enrolled experienced at least 4 seizures and 10% experienced at least 10 seizure episodes (37). Few of the children in either study ended up with intractable seizure (39).

## Factors which increase the recurrence risk

The underlying etiology and abnormal EEG are among those factors that elevate the risk of experiencing another seizure (40). The recurrence rate is higher in individuals who have a remote symptomatic etiology; in those with an idiopathic or cryptogenic etiology it is remarkably lower (38-41). By the term “remote symptomatic” it is meant without immediate cause but a prior identifiable major brain insult or accompanying conditions such as cerebral palsy or mental retardation. Idiopathic seizures are not associated with a known CNS defect and are of suspected genetic etiology, and cryptogenic seizures occur in individuals otherwise normal with no clear etiology (42).

For children with first seizure that are idiopathic/ cryptogenic, the recurrence risk is generally between 30 and 50% by 2 years (34) and for remote symptomatic seizures, the estimate of recurrence risk is generally above 50% (34, 36). An EEG performed after the first seizure also helps to predict recurrence (34, 43), particularly if there is an epileptiform abnormality (2).

## Special consideration if the first seizure is prolonged

Approximately 10 to 12 % of children and adults with a first unprovoked seizure will present with a seizure lasting at least half an hour (status epilepticus) (44). 

In the absence of an acute or progressive brain injury or disease, the morbidity and mortality of status epilepticus in children is relatively low (16). Of 46 children with “idiopathic” seizure, in a study of sequela of status epilepticus in 193 children, 2 children had mental retardation, but they had been studied retrospectively and details of the clinical circumstances were not clear. None of the children who were investigated prospectively had motor or cognitive problems (16). The overall recurrence risk following a prolonged first seizure was no different from the recurrence risk following a brief first seizure.

However, if a child with an initial prolonged seizure did experience a seizure recurrence, it was more likely to be prolonged again. Of 24 children with initial episodes of status epilepticus who had a recurrence, 5 (21%) had status epilepticus as a recurrence, whereas of 157 whose first seizures were brief and who had a recurrence, 2 (1%) had status epilepticus as their recurrence (2).

## The effectiveness of treatment after a first seizure in prevention of recurrence

There is one study which included solely the children randomized to treatment versus no treatment after a first nonfebrile seizure (45). In that study with a total of 31 children, 2 of 14 children (14%) treated with carbamazepine (CBZ), experienced a recurrence compared with 9 of 17 (53%) who were not treated. Although the recurrence rate up to 1 year was significantly lower in the treated group, only 6 of 14 (43%) patients randomized to CBZ completed the year with no significant side effect or seizure recurrence and 7 of 17 ( 41%) assigned to no medication had no seizure recurrence. One study in which 228 subjects were randomized to valproic acid or placebo included 33 adolescents between the ages of 16 and 19 (46). The follow up period for this trial was between 9 month and 5 years. Five (4%) of the treated group experienced a recurrence compared with 63 (56%) of those treated with placebo. In conclusion, different studies indicate children and adults who were assigned to treatment with AED after a first seizure had lower risk of seizure recurrence.

## What is the long term prognosis for seizure remission if a first seizure is treated?

One study had 419 subjects, of whom 114 were between 2 and 16 years of age. This study compared the probability in patients treated after a first seizure versus patients treated after a second seizure.

Follow up was for at least 3 years, with a minimum of 2 years seizure- free. Patients treated after the first seizure and those treated after a second seizure had the same probability of achieving a 1 or 2 year seizure remission (2, 47). In short, there is no evidence to prove a difference when treatment is started after the first seizure versus after a second seizure in achieving a 1 or 2 year seizure remission (2).

## The nature and frequency of side effects of AED therapy after a first seizure in children

AED therapy may cause systemic side effects such as skin rash, hirsutism, weight gain and menstrual problems. Dangreous reactions such as hepatic toxicity, bone marrow toxicity and Steven-Johnson syndrome cannot be predicted and early recognition is important. Side effects of AED occurring in children include harmful effects on cognition and higher cortical function which are often dose related and less recognized (48). If the patient is a teenage girl who may get pregnant, the risk of teratogenicity is another concern (49). 

Trials that report data relating to efficacy do not always include data relating to side effects. Data regarding toxicity or side effects of AED are not specifically available for treatment after a first seizure (2).

In a blinded, randomized, crossover study comparing Phenobarbital (PB) with Valproic Acid (VPA), children taking PB had lower scores on four tests of cognitive function and had more behavioral problems particularly hyperactivity, that were not dose related (50). In another study of children with newly diagnosed epilepsy in which 23 children received CBZ, 20 got PHT (phenytoine) and 21 used VPA, those on CBZ and PHT were slower on tests of information processing and children on CBZ showed increased irritability (51).

A series of studies each designed to compare the cognitive effects of low versus high levels of AED in children with epilepsy found no differences between low and high levels with either CBZ or PHT (52-53). A report from the American Academy of Pediatrics regarding general recommendations for awareness of behavioral and cognitive effects of AEDs noted that high blood levels of some AEDs (PHT, PB, Primidone) were significantly related to cognitive decline (48). 

Systemic side effects other than behavioral or cognitive side effects also occur in children placed on AED.

A randomized and blinded prospective study of 151 children with epilepsy found that 32% of children on PB, 19% of children on VPA, and 40% of children on PHT had more than one toxic side effect. Fifty-eight percent of those on PHT experienced gingival hyperplasia, and 25% had dose-related ataxia or sedation. Follow up was 2 years (54).

**In conclusion**, the American Academy of Neurology and the Practice Committee of the Child Neurology Society in their Practice Parameter made the following statement (2):

The majority of children who experience a first unprovoked seizure will have few or no recurrence; only approximately 10% will go on to have many (≥10) seizures regardless of therapy. Treatment with AED after a first seizure as opposed to after a second seizure has not been shown to improve prognosis for long-term seizure remission.

Treatment has been shown in several studies, combining both children and adults, to reduce the risk of seizure recurrence. There is a relative paucity of data from studies involving only children after a first seizure. AED therapy in children who have at least 2 seizures (epilepsy) has potential for inducing serious pharmacologic and psychosocial side effects. No separate data exist specifically for treatment side effects in children who have experienced only a single seizure. There is no evidence about whether treatment specifically after the first seizure alters the risk of sudden unexpected death in epileptic patients in children.

The two above mentioned organizations recommend the decision as to whether or not to treat with AED following a first unprovoked seizure in a child or adolescent must be based on a risk-benefit assessment that weighs the risk of another seizure (both the statistical risk of recurrence and the potential consequences of a recurrence) against the risk of chronic AED therapy (cognitive, behavioral and physical, as well as psychosocial). This decision must be individualized and take into account both medical issues and the patients’ and family’s preference.

The Two following recommendations are made for children and adolescents who have experienced a first seizure: 

1. Treatment with AED is not indicated for the prevention of the development of epilepsy

2. Treatment with AED may be considered in circumstances where the benefits of reducing the risk of a second seizure outweigh the risk of pharmacologic and psychosocial side effects.
